# Long-term results of lymphedema treatment with Combined lymph node transfer and collagen scaffolds: An Observational Study

**DOI:** 10.1016/j.jpra.2024.11.019

**Published:** 2024-12-02

**Authors:** Dimitrios Dionyssiou, Antonios Tsimponis, Eleni Georgiadou, Konstantina Mamaligka, Efterpi Demiri

**Affiliations:** Department of Plastic Surgery, Aristotle University of Thessaloniki, School of Medicine, Papageorgiou General Hospital, Thessaloniki, 54603, Greece

**Keywords:** Lymphedema microsurgical treatment, Lymph node transfer, Collagen scaffolds, Lymphagiogenesis

## Abstract

**Aim:**

Vascularized lymph node transfer (VLNT) accelerates growth factor secretion, lymphatic endothelial cell migration toward the interstitial flow and lymphagiogenesis in a multidirectional pattern. Our observational study aimed to examine the hypothesis that nanofibrillar collagen scaffolds (NCS) combined with VLNT can provide guided lymphagiogenesis creating long-lasting lymphatic pathways.

**Methods:**

Twenty-four patients (21 female, 3 male) underwent a lymphatic microsurgery for upper (*n* = 11) or lower (*n* = 13) limb secondary lymphedema and completed at least 18 months follow-up were selected and equally divided in 2 groups; Group-A underwent VLNT, Group-B underwent combined VLNT and NCS procedure. Lymph node flap sizes, harvesting procedure, and implantation location were similar in both groups. Demographics, lymphedema etiology and staging, limb volumetry, and somatometric data were recorded. Pre- and post-operative data for limb-volume difference, infection episodes/year, and indocyanine-green (ICG) lymphography changes were documented in all patients.

**Results:**

Mean follow-up was period was 42 months (24–60 months) in Group-A, and 27 months (18–48 months) in Group-B patients. Demographic data, lymphedema etiology, and staging were comparable in both groups. Pre- and post-operative edema volume difference for Group-A was 36 % and 25 % (*p* < 0.001), and 33 % and 14 % in Group-B (*p* = 0.001), respectively. The mean number of infection episodes decreased in Group-A and B from 1.75 to 0.33 and from 2.17 to 0.42 per patient/year, respectively. ICG mean stage in Group-A was 3.58 pre- and 3 post-operatively (*p* = 0.045), and 3.67 pre- and 2.08 post-operatively in Group-B (*p* = 0.506). A statistically significant difference was found in post-operative volume difference between the 2 groups (*p* = 0.008) and post-operative ICG changes (*p* < 0.001). ICG-lymphography demonstrated new lymphatic vessel formation at the NCS implantation location.

**Conclusions:**

Long-term follow-up of the patients treated using combined VLNT-NCS approach revealed a statistically significant improvement regarding volume reduction, infection episodes per year, ICG downstaging, and new lymphatic vessel formation, compared to VLNT alone.

## Introduction

Lymphedema is the abnormal accumulation of protein-rich lymphatic fluid in the interstitial tissue of the affected areas, mainly the extremities, due to lymphatic drainage dysfunction. The subsequent chronic inflammation, fibrosis, impaired local immunity, and limb disfigurement may cause local pain, disability, frequent infections, and even psychosocial issues.^1^, ^2^, ^3^, ^4^

The 2 main lymphedema etiologies include primary and secondary lymphedema. Primary lymphedema includes inherited or congenital conditions, most often associated with genetic mutations. Secondary lymphedema occurs due to an acquired insult to the lymphatic system. In developed countries, most cases are related to oncologic surgeries (e.g., breast, prostate, and gynecological cancers), or therapeutic interventions, such as lymphadenectomy, chemotherapy, and radiation.^3^^,^^5^

The management of lymphedema can be conservative, including manual lymphatic drainage, bandaging, compression garments,^6^ and/or surgical.^7^ Although conservative techniques remain the initial approach,^8^ microsurgical procedures, especially vascularized lymph node transfer (VLNT)^9^^,^^10^ and lymphatic-venous anastomoses (LVA)^11^ are being used in an increased fashion.^8^ VLNT appears to be particularly promising because revascularization improves transplanted lymph node survival and function.^12^ Healthy lymph nodes harvested from distant areas are transferred as free flap to the lymphedematous extremity to drain the lymph via lymphovenous connections and neolymphagiogenesis.^13^ This technique significantly reduces the volume difference between the limbs and infection episodes, while improving the quality of life by reducing compression garment usage and complication rates.^14^ The adjunctive implementation of aligned nanofibrillar collagen scaffolds (NCS) (BioBridge®—Fibralign Corp, Union City, CA, USA) appears to further enhance and sustain lymphagiogenesis and lymphatic drainage.^15^

BioBridge® NCS consist of parallel aligned porcine type I atelocollagen nanofibers.^13^^,^^16^ Their similar structure to the natural arrangement of human collagen fibers in connective tissue, promotes endothelial cell migration, attachment, and outgrowth,^13^ as reported by Nakayama et al., who tested arteriogenesis in the context of peripheral arterial disease.^17^ The role of NCS in lymphagiogenesis and lymph drainage was mostly confirmed in animal models^13^^,^^15^^,^^18^ and only recently in clinical series.^19^^,^^20^

This retrospective observational study aimed to evaluate the long-term results of combined VLNT and NCS implementation in patients with secondary lymphedema in comparison with standard VLNT procedure.

## Patients and methods

The present study followed the strengthening the reporting of observational studies in epidemiology (STROBE) guidelines^21^ and was conducted in accordance with the Declaration of Helsinki on investigation in humans.^22^ The study underwent an Institutional Review Board approval and all patients consented to participating in the study.

Twenty-four patients (21 women, 3 men) treated with lymphatic microsurgery for upper (*n* = 11) or lower (*n* = 13) limb secondary lymphedema and completed at least 18 months follow-up were included in this study and divided in 2 groups; Group-A patients underwent VLNT, whereas Group-B patients underwent combined VLNT and NCS procedure. The initial diagnosis was based on the patient's history and clinical examination, while lymphedema stage and characteristics were confirmed and classified according to the clinical findings**,** technetium-99 (Tc-99) nanocolloid Single Photon Emission Computed Tomography with a Computed Tomography (SPECT-CT) scan lymphoscintigraphy, and indocyanine-green fluorescent lymphography (ICG).^23^ VLNT with or without NCS procedures were performed between 2018 and 2022 by the same surgical team. Inclusion criteria involved patients with unilateral upper or lower limb secondary lymphedema uncured with conservative treatment, who were willing to undergo VLNT±NCS procedure and were able to undergo a long-term follow-up. Exclusion criteria were bilateral lymphedema, primary lymphedema, active or metastatic cancer, and unwillingness to participate in the study. The decision for NCS addition at the VLNT was based on the patients’ preference or insurance reimbursement.

Group-A consisted of 11 women and 1 man, with a mean age of 48.92 years (range 21–63 year) and a mean body mass index (BMI) of 28.02 kg/m^2^ (range 21.1–33.7 kg/m^2^). Group-B consisted of 11 women and 1 man, with a mean age of 50.42 years (range 24–62) and mean BMI of 26.07 kg/m^2^ (range 17.6–32.8 kg/m^2^). Etiologies of secondary lymphedema in both groups were almost similarly related to uterine, breast, and prostate cancer treatment and trauma. No patients had a history of previous lymphedema surgery (Table 1).

**Table 1:** Data summarizing the patients’ demographics, lymphedema etiology, location, and stage.

No	Gender	Age, years	BMI, kg/m^2^	Etiology	Location	ISL Stage pre-op	ICG Stage pre-op
Group A							
1	F	38	31.3	Breast cancer	LUL	3	3
2	F	42	29.2	Breast cancer	LUL	2	3
3	F	59	27.6	Uterine cancer	LLL	2	4
4	M	63	26.3	Prostate cancer	RLL	2	4
5	F	63	27.1	Uterine cancer	RLL	2	3
6	F	56	30.6	Uterine cancer	LLL	3	4
7	F	59	24.2	Uterine cancer	LLL	2	4
8	F	38	31.4	Uterine cancer	LLL	3	4
9	F	21	33.7	Trauma	LLL	3	4
10	F	51	27.9	Breast cancer	RUL	2	3
11	F	47	25.9	Breast cancer	RUL	2	4
12	F	50	21.1	Breast cancer	RUL	2	3
Mean		48.92	28.02			2.33	3.58
Group B							
1	F	48	32.2	Breast cancer	LUL	3	4
2	F	50	31.3	Breast cancer	LUL	2	3
3	F	61	28.2	Uterine cancer	RLL	3	4
4	F	57	32.8	Uterine cancer	RLL	3	4
5	F	48	21	Uterine cancer	RLL	2	4
6	F	59	23.4	Uterine cancer	LLL	2	3
7	F	40	26.8	Uterine cancer	LLL	2	4
8	F	62	22.8	Uterine cancer	LLL	2	4
9	M	24	22.7	Trauma	LLL	2	4
10	F	53	28.5	Breast cancer	RUL	2	4
11	F	55	25.6	Breast cancer	RUL	2	3
12	F	48	17.6	Breast cancer	RUL	2	3
Mean		50.42	26.07			2.25	3.67

F, female; M, male; BMI, body mass index; RLL, right lower limb; LLL, left lower limb; RUL, right upper limb; LUL, left upper limb; ISL, International Society of Lymphology; ICG, indocyanine green fluorescent lymphography.

Volume differences and infection episodes (erysipelas or lymphangitis) occurring on the lymphedematous extremity per patient per year were recorded, in addition to the lymphedema staging and ICG findings. A truncated cone formula based on 4-cm intervals serial perimeter measurements was used to measure the volume of the affected and contralateral limbs.^24^ Their difference representing the excess volume of the affected limb was expressed as the percentage of the volume of the unaffected limb (V %). Before treatment, lymphedema was classified as ICG stage III in 5 cases, and stage IV in 7 cases of Group- A, whereas ICG stage III in 4 cases, and stage IV in 8 cases of Group- B (Table 1).

Volumetric measurements, infection episodes per year, and lymphedema staging were recorded and photo-documented pre- and post-operatively (Table 2).

**Table 2:** Volumetric and clinical data of the patients.

No	V% pre-op	V% post-op	I pre-op	I post-op	ICGS pre-op	ICGS post-op	New lymphatic vessels at NCS area	Follow-up (months)
Group A								
1	48	37	2	0	3	3	N/a	24
2	27	18	0	0	3	2	N/a	24
3	32	22	3	1	4	4	N/a	36
4	37	24	0	0	4	3	N/a	48
5	22	14	4	0	3	3	N/a	48
6	47	36	1	0	4	3	N/a	60
7	63	49	0	0	4	3	N/a	48
8	43	21	1	0	4	3	N/a	36
9	48	31	7	2	4	4	N/a	48
10	22	16	1	1	3	3	N/a	36
11	31	17	2	0	4	3	N/a	60
12	17	9	0	0	3	2	N/a	36
Mean	36	25	1.75	0.33	3.58	3		42
Group B								
1	52	24	2	0	4	2	YES	24
2	35	12	0	0	3	2	N/a	18
3	46	17	1	0	4	2	YES	24
4	43	19	1	0	4	2	YES	24
5	42	20	0	0	4	2	YES	18
6	15	4	3	1	3	2	YES	24
7	36	17	1	0	4	2	N/a	24
8	31	14	8	2	4	2	N/a	24
9	35	7	4	0	4	2	YES	24
10	30	12	5	1	4	3	YES	36
11	21	9	0	0	3	2	N/a	48
12	4	8	1	1	3	2	YES	36
Mean	33	14	2.17	0.42	3.67	2.08		27

V, volume (% difference of edema); I, number of infections (episodes per patient per year); ICGS, stage according to indocyanine green fluorescent lymphangiography, NCS, nanofibrillar collagen scaffolds.

### Statistical analysis

Statistical analysis was conducted to evaluate the differences in volumetric measurements and infection rates among patients who were administered 2 distinct therapeutic interventions: VLNT vs. VLNT+NCS). The Shapiro-Wilk test was applied to assess the normality of distribution for all parameters within each cohort. Depending on the nature of the variables under consideration—whether parametric or non-parametric—the paired samples *t*-test, independent samples *t*-test, and Mann–Whitney U tests were employed to discern the statistically significant differences in volumetric measurements, the number of infection episodes and ICG staging preoperatively and post-operatively. The alpha level for determining statistical significance was preset at a p-value of <0.05.

### Surgical techniques

Group-A patients underwent VLNT procedure. Group-B patients underwent VLNT combined with insertion of 5 or 10 NCS, for the management of upper or lower limb lymphedema, respectively (Table 3).

**Table 3:** Summary of surgical procedures of the patients.

No	Previous lymphedema surgery	Current lymphedema surgery	Number of NCS
Group A			
1	N/a	VLNT at left axilla	0
2	N/a	VLNT at left axilla	0
3	N/a	VLNT at left groin	0
4	N/a	VLNT at right groin	0
5	N/a	VLNT at RLL upper tibia/GA	0
6	N/a	VLNT at LLL lower tibia/PTA	0
7	N/a	VLNT at left groin	0
8	N/a	VLNT at left groin	0
9	N/a	VLNT at LLL upper tibia/GA	0
10	N/a	VLNT at right axilla	0
11	N/a	VLNT at right axilla	0
12	N/a	Pedicle propeller VLNT	0
Group B			
1	N/a	VLNT at left axilla+NCS	5
2	N/a	VLNT at left axilla+NCS	5
3	N/a	VLNT at right groin+NCS	10
4	N/a	VLNT at lower tibia/ PTA+NCS	10
5	N/a	VLNT at RLL upper tibia/ GA+NCS	10
6	N/a	VLNT at LLL lower tibia/ PTA+NCS	10
7	N/a	VLNT at left groin+NCS	10
8	N/a	VLNT at left groin+NCS	10
9	N/a	VLNT at LLL upper tibia/GA, Propeller VLNT at left groin+BB	10
10	N/a	VLNT at right axilla+NCS	5
11	N/a	VLNT at left axilla+NCS	5
12	N/a	Pedicle propeller VLNT+NCS	5

VLNT, lymph node transfer; N/a, non-applicable; RLL, right lower limb; LLL, left lower limb; PTA, posterior tibial artery; GA, gastrocnemius artery; NCS, nanofibrillar collagen scaffolds.

In patients with upper limb lymphedema, the lymph node flaps were harvested according to the selected lymph node technique^25^ from the inguinal area and transplanted to the axilla in 8 Group-A and B participants, whereas in 2 cases, a pedicled propeller lymph node flap was elevated from the lateral thoracic area and transferred to the ipsilateral axilla.

In all lower limb lymphedema cases, the preferred donor site of the lymph node flap was the lateral thoracic area, whereas the recipient site was chosen according to the ICG and SPECT-CT lymphoscintigraphy findings. If the lymphangiographies indicated a sufficient lymphatic presence at the groin, the flap was transplanted at the inguinal area to augment lymphagiogenesis. In cases of advanced lymphedema with poor or no lymphatic circulation at the thigh region, the flap was placed at the proximal or distal tibial area, according to the level of lymphatic functionality. Recipient vessels were branches of the posterior tibial, gastrocnemius, or femoral artery and their neighboring veins, while all flaps were anastomosed in an end-to-end fashion and placed in a way to continue the axiality of the lymphatic vessels. In trauma-related lymphedema patients, the free lymph node flap was transplanted to the tibial area, and a second pedicled propeller inguinal VLNT was transferred to the groin area.

**Figure 1:**
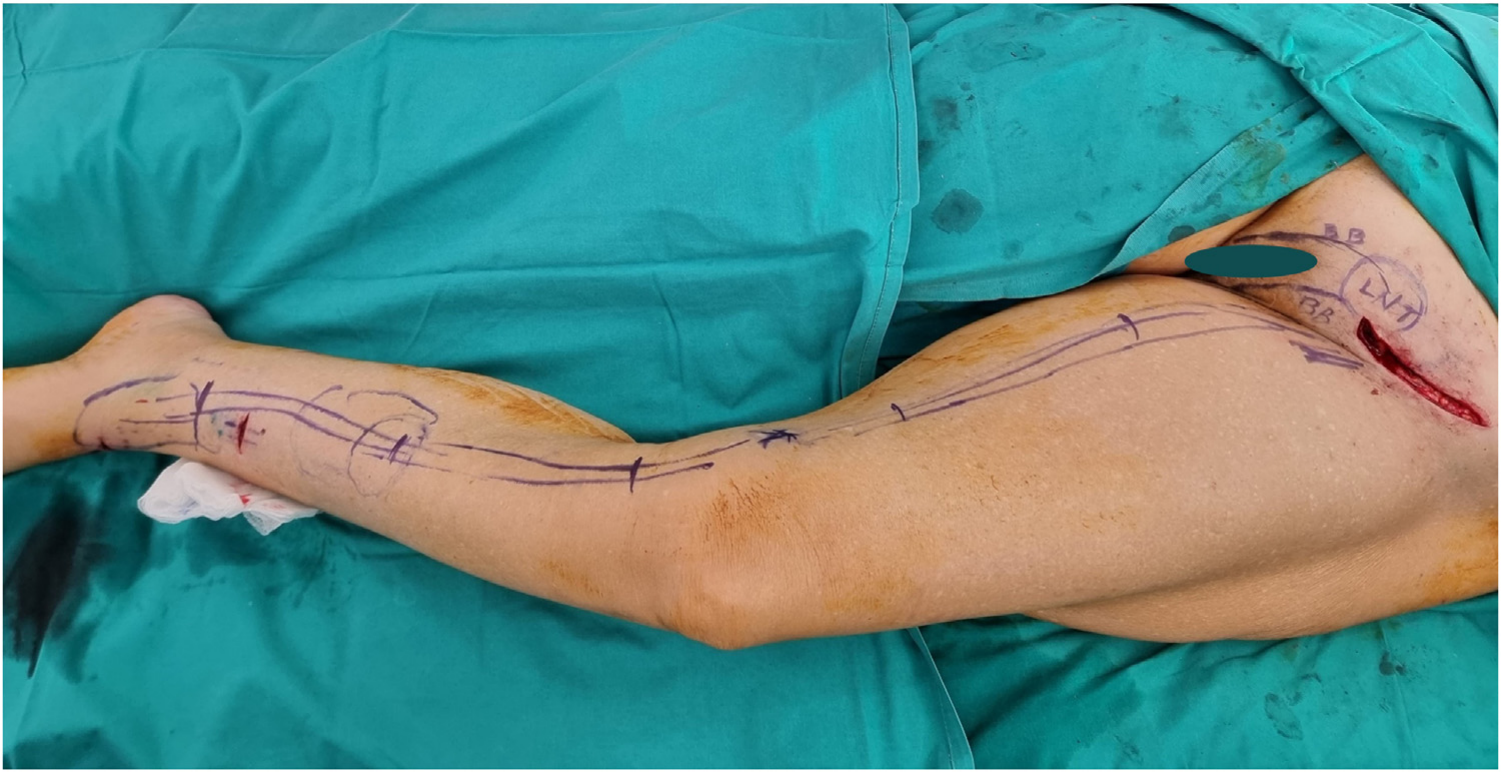
Illustrations showing the area of lymph node flap transfer at the groin (LNT), and BioBridge® NCS insertion at the subdermal level along the blue lines (inner area of the limb).

**Figure 2:**
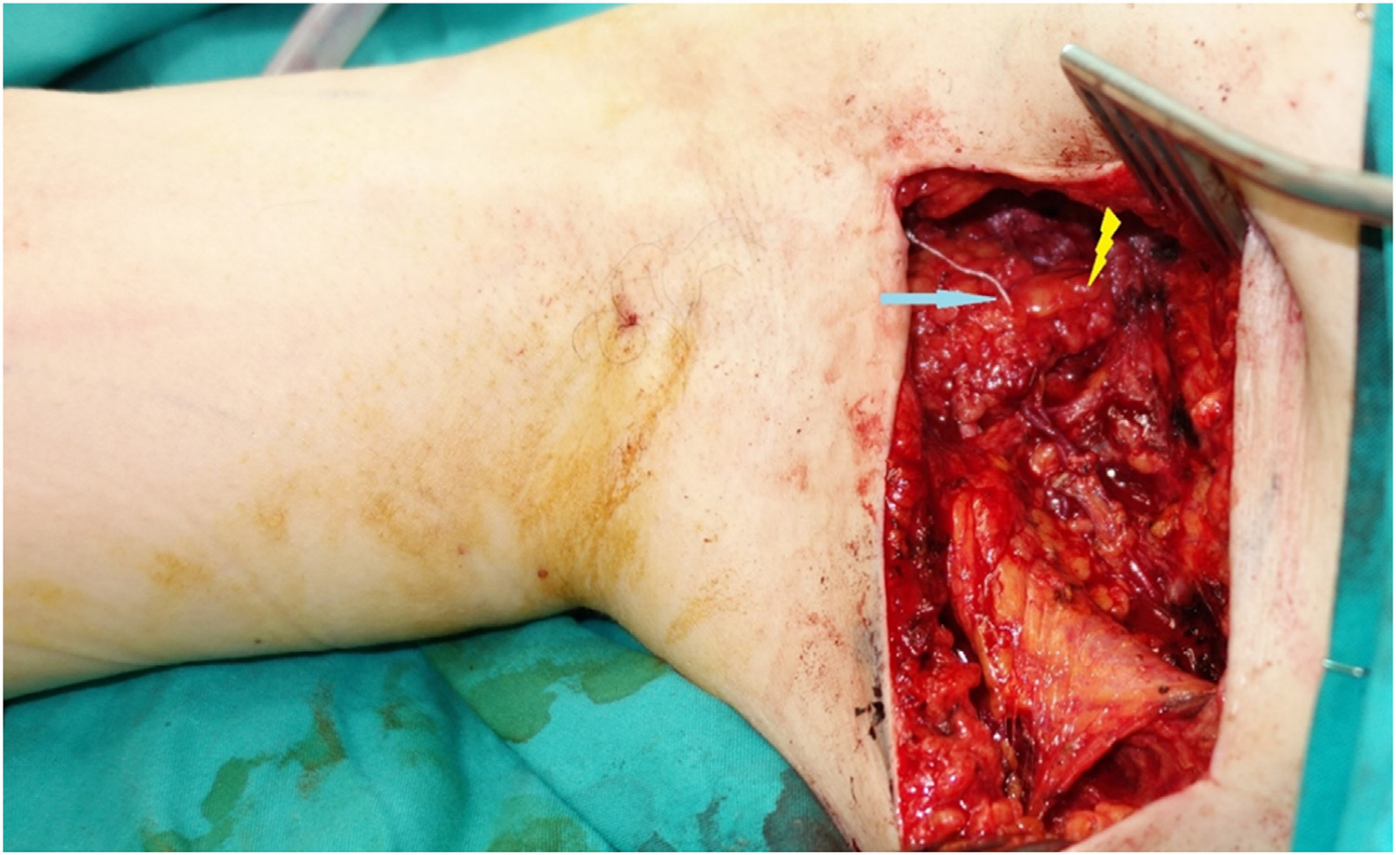
Inserted NCS (blue arrow) at the axilla on the upper part of the vascularized lymph node flap (yellow thunderbolt).

**Figure 3:**
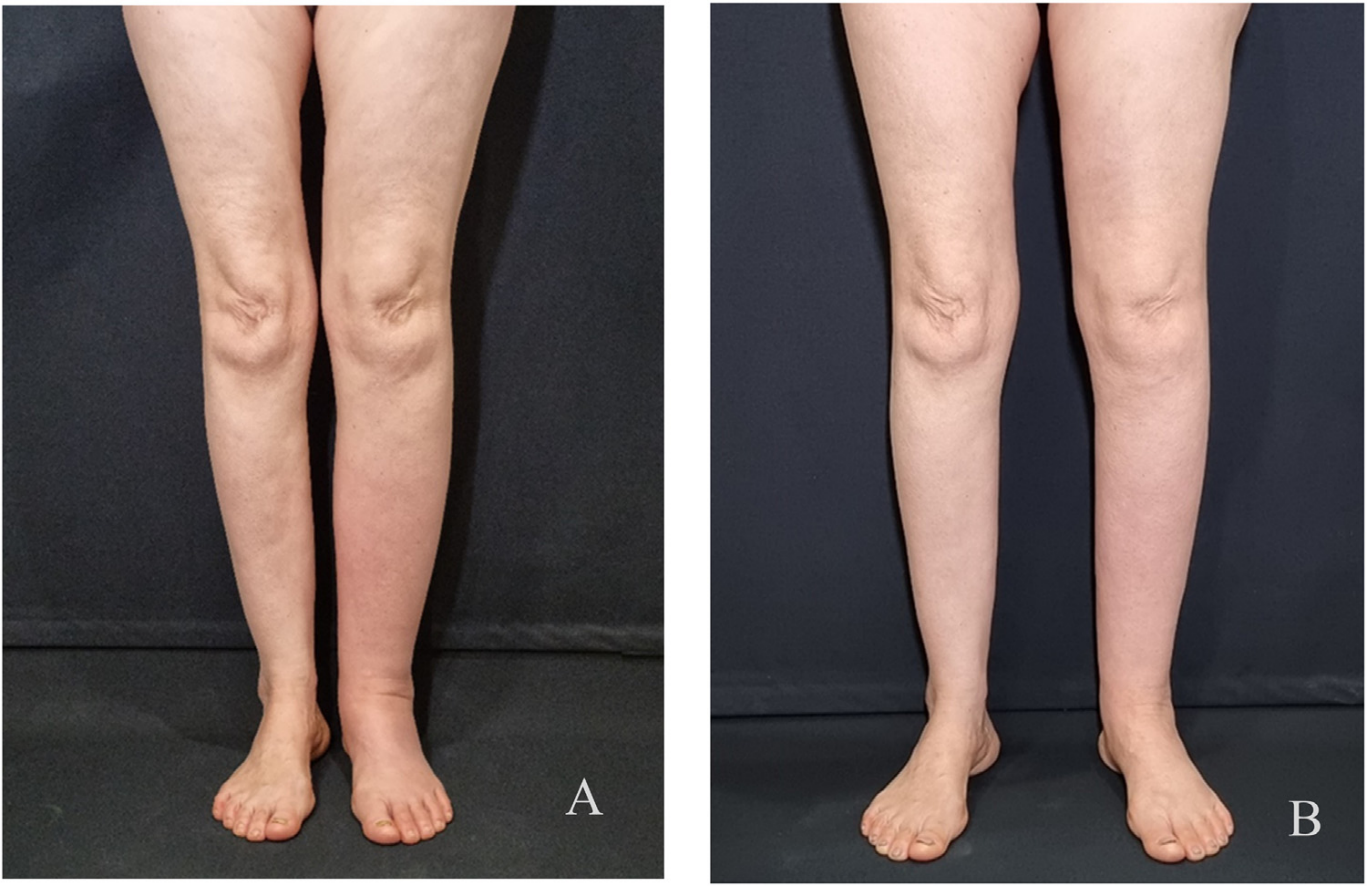
Preoperative photograph of a 59-year-old female patient with secondary left lower limb lymphedema with 15 % volume difference and history of 3 episodes of infection per year (A). Result at 12 months postoperatively, with a 4 % volume difference; only one infection episode was reported after surgery (B).

BioBridge® NCS were inserted subdermally in the continuity, one next to the other (Figure 1) using a sharp suture-grasper device. The first collagen scaffold was placed from the lymph node flap site to the periphery and attached to the flap with a micro ligating titanium clip (Figure 2). The length of each collagen scaffold (approximately 15 cm long) was drawn onto the skin of the insertion area to indicate the distal insert point of the suture-grasper to place the second NCS. Therefore, the exit point of the first scaffold was always used as the entry point for the second scaffold, as shown in Figure 1. In all cases, with either upper or lower limb lymphedema, collagen scaffolds were placed along the most affected lymphedematous areas, aiming to provide lymphagiogenetic pathways and enhance future lymphatic connections between the transplanted lymph nodes and healthy lymphatic vessel of the area. Based in our experience and due to the fibrotic lymphedema tissue, scaffolds remain in place without the need of extra suturing (Figure 3).

### Pre-operative and post-operative manual lymphatic drainage regime

All participants received 5 to 10 sessions of manual lymphatic drainage and bandaging preoperatively and another 10 sessions post-operatively, followed using compression garments class II for upper or class III for lower limb for a period of 6 to 12 months, and completed the preoperative and post-operative regime.

## Results

All patients conformed well to the offered treatment, and no major post-operative complications, such as wound dehiscence, infection, or donor site lymphedema, were observed. Τhe follow-up time ranged from 24 to 60 months (mean 42 months) for Group- A and from 18 to 48 months (mean 27 months) for Group- B. Post-operative SPECT-CT lymphoscintigraphy showed functional activity of the implanted lymph nodes or improvement of the dermal back flow in 9 patients of Group-A and similar in Group-B.

**Figure 4:**
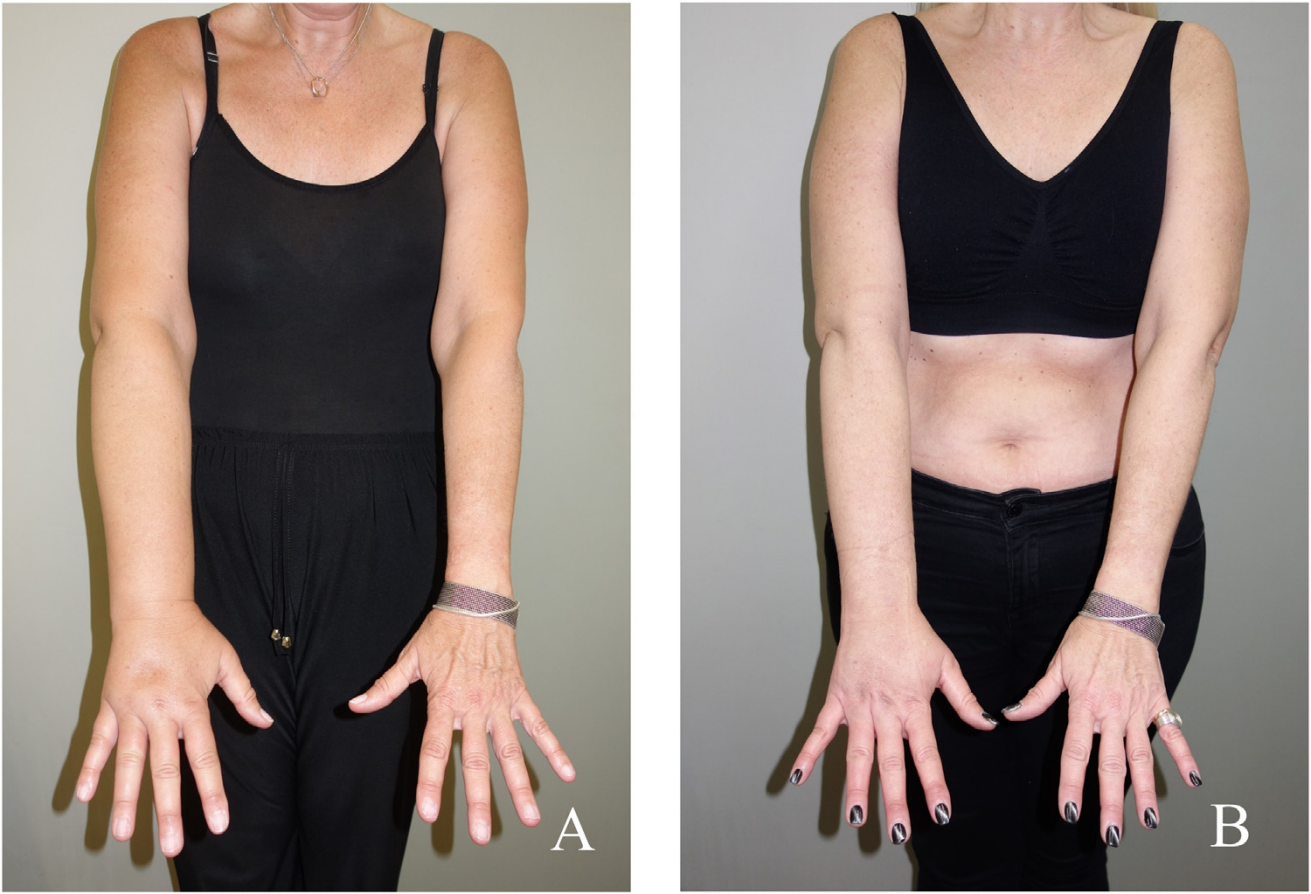
Preoperative photograph of a 55-year-old female patient with secondary right upper limb lymphedema with 21 % volume difference (A). Result at 12 months post-operative of a combined LNT+NCS procedure, with a 9 % volume difference (B).

Table 4 summarizes the values concerning the volume differences between limbs, the infection episodes and the ICG-based lymphedema staging, preoperatively and at the final documented follow-up. Our results showed that the volume of the affected limb was significantly decreased after treatment in both groups (Group-A *p* < 0.001 and Group-B *p* ≤ 0.001) (Table 4), but the mean volume reduction was significantly higher in Group-B compared to Group- A patients (11% vs. 19 %, *p* = 0.028) (Table 5), indicating superior post-treatment results in Group-B (Figure 4). Regarding the infection episodes and ICGS, the evaluation of each treatment showed a bigger mean difference in infection rate in Group-B (1.75) compared to Group-A (1.42), as well as in ICGS (Group- B: 1.59 vs. Group- A: 0.58).

**Table 4:** Correlation of mean volume reduction, infection rate, and ICGS of the affected extremity in groups A and B, pre- and post-operative of any treatment.

Group	Mean volume pre-op	Mean volume post-op	p
A	36	25	<0.001
B	33	14	<0.001

Group	Mean infection rate pre op	Mean infection post-op	p

A	1.75	0.33	0.014
B	2.17	0.42	0.01

Group	Mean ICGS pre-op	Mean ICGS post-op	p

A	3.58	3	0.02
B	3.67	2.08	<0.001

ICGS, stage according to indocyanine green, fluorescent lymphangiography.

**Table 5:** Differences in the affected extremity volume and infection rate in groups A and B.

Differences	Group A	Group B	p
Mean difference in volume	11	19	0.028
Mean difference in infection rate	1.42	1.75	0.659
Mean difference in ICGS	0.58	1.59	<0.001

**Figure 5:**
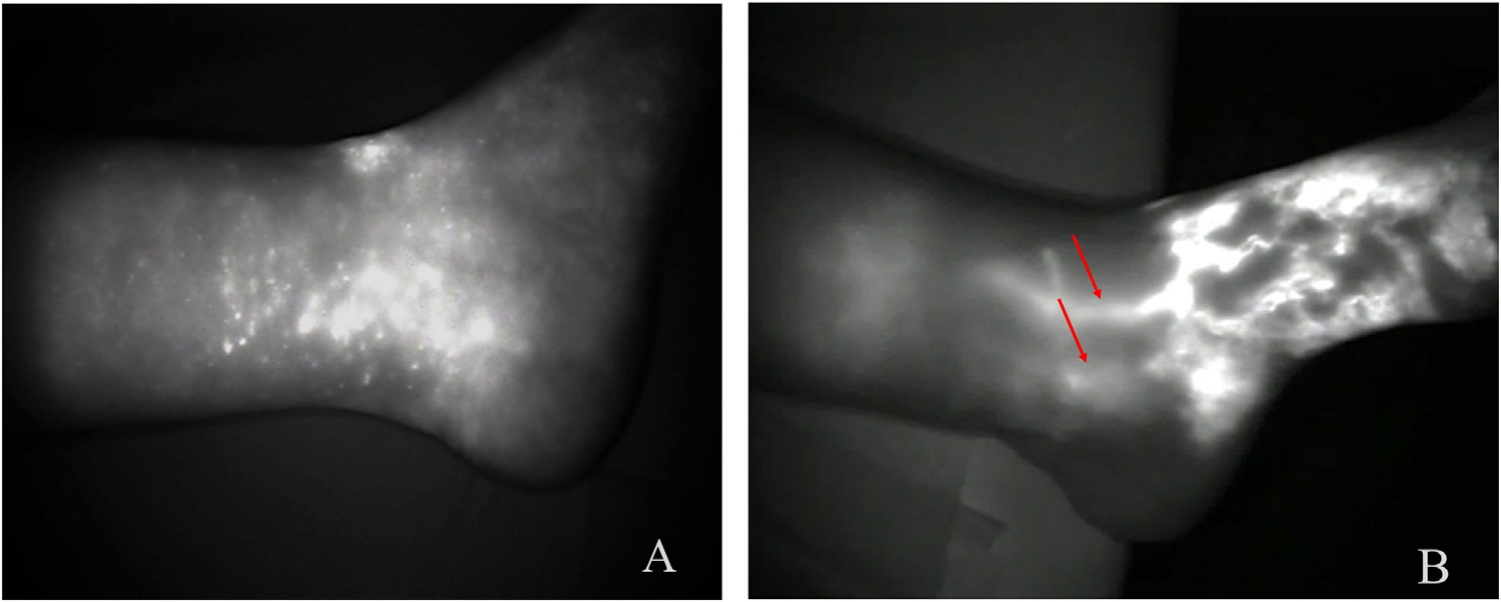
Preoperative ICG showing a diffuse and star-dust dermal back-flow pattern in the lymphedematous leg (A), while at the 12 months follow-up a downstaging to a splash pattern is documented (B) with the presence of neolymphatic vessels (red arrows).

ICG showed lymphedema downstaging of at least one level and formation of new lymphatic vessels in the location of NCS implantation in Group-B cases (Figure 5). The best results regarding neolymphangiogenesis at the area of NCS implantation were related to a two-level downstaging of the operated lymphedematous limb (5 out of 9 cases).

Analyzing the results of the 2 therapeutic modalities in patients with secondary lymphedema, the combined VLNT and NCS procedure was significantly proven to be more effective (*p* = 0.028) compared to VLNT alone (Table 5).

## Discussion

Lymphedema, regardless of its etiology, remains a challenging disease to cure, having a negative effect on the patient's health and quality of life. Although several treatment strategies have been proposed, the combination of conservative and surgical approaches appears to provide better results.^26^ The expanding applications of microsurgical techniques, and particularly VLNT, have been reported to provide very satisfying outcomes, regarding volume and infection episode reduction.^27^ Reported rates of volume reduction following VLNT are typically <60 %.^27^ Indeed, in our previous retrospective cross-sectional study on using VLNT for treating upper extremity breast cancer-related lymphedema, volume reduction was documented as 55.7 %, while a significant infection episode reduction was also reported, i.e., from 1.94 to 0.27 episodes per year, pre- and post-operatively, respectively.^28^ In another comparative clinical study, VLNT was used for patients with secondary lymphedema, neither the stage of lymphedema nor vascular pedicle of the lymph node flap used (superficial inferior epigastric artery, superficial circumflex iliac artery, or a perforator branch) was reported to have a significant impact on limb-volume reduction, while better outcomes were associated with larger lymph node flaps.^29^ Similarly, mean upper extremity volume reduction was reported to be 57 % after combining conservative therapy and VLNT from the groin area, based on the superficial inferior epigastric artery and superficial circumflex iliac artery. Nguyen et al. performed free vascularized omental lymphatic flap transfer for upper and lower limb lymphedema with a mean of 22 % volume improvement and great resistance to infections.^30^ In addition, Moon et al. studied the effectiveness of VLNT and post-operative application of compression garments for at least 6 months. In 37 cases of upper limb lymphedema and 34 cases of lower limb lymphedema, they reported a mean rate of 29.4 % and 36.1 % volume difference between the affected and unaffected extremities, respectively; in this study, groin flaps incorporating superficial inguinal lymph nodes were used for upper limb lymphedema, whereas lateral thoracic lymph node flaps incorporating axilla level lymph nodes were used for lower limb lymphedema.^31^ Our preferred lymphatic flap for upper limb lymphedema remains the inguinal donor area based on the SPECT-CT examination. In selected cases, even after axillary lymphadenectomy and radiotherapy, when the lateral thoracic lymph nodal flap can be identified, a propeller like local flap can augment the lymphatic circulation of the axilla. For the lower extremity lymphedema, our VLNT donor side first choice, based on our experience and unpublished SPECT-CT findings, remain the free lateral thoracic flap based on the lateral thoracic vessels or branches of the thoracodorsal vessels.

In an effort to further improve the outcomes following VLNT, aligned NCS have been shown to promote lymphagiogenesis and reestablishment of lymphatic drainage when implanted adjunctively to autologous lymph node transfer. Most studies to date have involved animal models of lymphedema.^13^^,^^15^^,^^16^ Nguyen et al. reported 50 % volume reduction after BioBridge® implantation as a secondary procedure in previously operated VLNT patients.^18^ Moreover, in a retrospective cohort study, the same authors found a 121.3 ± 13.2 % volume reduction in the subgroup that underwent VLNT and BioBridge® implantation versus 56.25±30.8 % (*p* = 0.026) in the group that only received a standard VLNT. Post-operative lymphatic mapping with ICG following BioBridge® placement confirmed the presence of significantly increased new lymphatic collectors and decreased dermal backflow.^19^ Deptula et al. in a multimodal approach of upper and lower extremities’ lymphedema, reported a 103 % (±31 %) volume reduction after liposuction, VLNT, and NCS placement.^20^

In our reported observational study with patients of different etiology or stages, with upper or lower limb lymphedema, combined lymph node transfer and collagen scaffold operation was compared to lymph node transfer alone. The number of collagen threads was 5 for upper or 10 for lower limb lymphedema, having as a main target to create a continuity of scaffolds at the whole length of the limb.

A long-term follow-up, has shown bigger volume reduction, decreasing the infection episodes per year and downstaging the lymphedema level in patients who received VLNT and NCS. Improvement in the superficial lymphatic circulation and presence of new lymphatic vessels around the implanted collagen scaffolds were also observed. Although there are some study limitations, including small number of patients, various etiologies, and associated surgeries, the results are very encouraging. Furthermore, if we analyze each subgroup of patients separately, based on the lymphedema stage, etiology, or operated limb (upper/lower extremity), the outcomes regarding clinical and imaging lymphedema improvement might appear even more encouraging. Evidently, larger clinical series and more homogenous populations are needed to draw firmer conclusions.

## Conclusions

Our long-term follow-up study documents a bigger volume reduction of upper and lower limb lymphedema, as well as a bigger decrease in soft tissue infections when a combined surgical approach with VLNT and nonofibrilar collagen scaffold implantation is applied. In addition, new lymphatic vessels have been shown to grow around the implanted scaffolds, in areas where there was previously no lymphatic appearance.

## Authors’ contributions

Made substantial contributions to the conception and design of the study: Dionyssiou D, Demiri E. Performed data acquisition, analysis and interpretation of data for the work: Dionyssiou D, Tsimponis A, Georgiadou E, Mamaligka K, Demiri E. All authors helped with drafting or revision of the manuscript for important intellectual content and provided final approval of the version to be published and agreed to be accountable for all aspects of the work in ensuring that questions related to the accuracy or integrity of any part of the work are appropriately investigated and resolved.

Dimitrios Dionyssiou

Efterpi Demiri

Antonios Tsimponis

Eleni Georgiadou

Konstantina Mamaligka

All authors should have made substantial contributions to **all** of the following:

(1) The conception and design of the study, or acquisition of data, or analysis and interpretation of data
(2) Drafting the article or revising it critically for important intellectual content
(3) Final approval of the version to be submitted.

## Availability of data and materials

All data are included, shared, and explained in the submitted manuscript.

## Financial support and sponsorship

None.

## Conflicts of Interest

“All authors declared that there are no conflicts of interest.”

## References

[1] Alitalo K. (2011). The lymphatic vasculature in disease. Nat Med.

[2] Grada A.A., Phillips T.J. (2017). Lymphedema: pathophysiology and clinical manifestations. Am Acad Dermatol.

[3] Lyons O.T.A., Modarai B. (2013). Lymphoedema. Surg.

[4] Fu M.R., Axelrod D., Haber J. (2008). Breast-Cancer-related lymphedema: information, symptoms, and risk-reduction behaviors. J Nurs Scholarsh.

[5] Sleigh B.C., Manna B. (2022). StatPearls.

[6] Lawenda B.D., Mondry T.E., Johnstone P.A.S. (2009). Lymphedema: a primer on the identification and management of a chronic condition in oncologic treatment. CA Cancer J Clin.

[7] Merchant S.J., Chen S.L. (2015). Prevention and management of lymphedema after breast cancer treatment. Breast J.

[8] Carl H.M., Walia G., Bello R. (2017). Systematic review of the surgical treatment of extremity lymphedema. J Reconstr Microsurg.

[9] Becker C., Hidden G. (1988). Transfer of free lymphatic flaps. Microsurgery and anatomical study. J Mal Vasc.

[10] Granzow J.W., Soderberg J.M., Kaji A.H., Dauphine C. (2014). An effective system of surgical treatment of lymphedema. Ann Surg Oncol.

[11] Boccardo F., Casabona F., De Cian F. (2009). Lymphedema microsurgical preventive healing approach: a new technique for primary prevention of arm lymphedema after mastectomy. Ann Surg Oncol.

[12] Cornelissen A.J., Qiu S.S., Lopez Penha T. (2017). Outcomes of vascularized versus non-vascularized lymph node transplant in animal models for lymphedema. Review of the literature. J Surg Oncol.

[13] Yang C.Y., Tinhofer I.E., Nguyen D., Cheng M.H. (2022). Enhancing lymphangiogenesis and lymphatic drainage to vascularized lymph nodes with nanofibrillar collagen scaffolds. J Surg Oncol.

[14] Winters H., Tielemans H.J.P., Paulus V., Hummelink S., Slater N.J., Ulrich D.J.O. (2022). A systematic review and meta-analysis of vascularized lymph node transfer for breast cancer-related lymphedema. J Vasc Surg Venous Lymphat Disord.

[15] Hadamitzky C., Zaitseva T.S., Bazalova-Carter M. (2016). Aligned nanofibrillar collagen scaffolds - Guiding lymphangiogenesis for treatment of acquired lymphedema. Biomaterials.

[16] Dionyssiou D., Nguyen D., Topalis A. (2024). Treatment of rat lymphedema by propeller lymphatic tissue flap combined with nano-fibrillar collagen scaffolds. J Reconstr Microsurg.

[17] Nakayama K.H., Hong G., Lee J.C. (2015). Aligned-Braided nanofibrillar scaffold with endothelial cells enhances arteriogenesis. ACS Nano.

[18] Nguyen D., Zaitseva T.S., Zhou A. (2022). Lymphatic regeneration after implantation of aligned nanofibrillar collagen scaffolds: preliminary preclinical and clinical results. J Surg Oncol.

[19] Nguyen D.H., Zhou A., Posternak V., Rochlin D.H. (2021). Nanofibrillar collagen scaffold enhances edema reduction and formation of new lymphatic collectors after lymphedema surgery. Plast Reconstr Surg.

[20] Deptula P., Zhou A., Posternak V., He H., Nguyen D. (2022). Multimodality approach to lymphedema surgery achieves and maintains normal limb volumes: a treatment algorithm to optimize outcomes. J Clin Med.

[21] von Elm E., Altman D.G., Egger M., Pocock S.J., Gøtzsche P.C., Vandenbroucke J.P (2008). STROBE Initiative, “The Strengthening the Reporting of Observational Studies in Epidemiology (STROBE) statement: guidelines for reporting observational studies. J Clin Epidemiol.

[22] World Medical Journal, 2008. [Online]. Available: https://www.wma.net/policies-post/wma-declaration-of-helsinki/.

[23] Yamamoto T., Yamamoto N., Doi K. (2011). Indocyanine green-enhanced lymphography for upper extremity lymphedema: a novel severity staging system using dermal backflow patterns. Plast Reconstr Surg.

[24] Brorson H., Höijer P. (2012). Standardised measurements used to order compression garments can be used to calculate arm volumes to evaluate lymphoedema treatment. J Plast Surg Hand Surg.

[25] Dionyssiou D., Demiri E., Sarafis A., CO Goula, Tsimponis A., Arsos G. (2019). Functional lymphatic reconstruction with the “Selected Lymph Node” technique guided by a SPECT-CT lymphoscintigraphy. J Surg Oncol.

[26] Finnane A., Janda M., Hayes S.C. (2015). Review of the evidence of lymphedema treatment effect. Am J Phys Med Rehabil.

[27] Rochlin D.H., Inchauste S., Zelones J., Nguyen D.H. (2020). The role of adjunct nanofibrillar collagen scaffold implantation in the surgical management of secondary lymphedema: review of the literature and summary of initial pilot studies. J Surg Oncol.

[28] Dionyssiou D., Demiri E., Tsimponis A. (2016). A randomized control study of treating secondary stage II breast cancer-related lymphoedema with free lymph node transfer. Breast Cancer Res Treat.

[29] Dionyssiou D., Sarafis A., Tsimponis A., Kalaitzoglou A., Arsos G., Demiri E. (2021). Long-Term outcomes of lymph node transfer in secondary lymphedema and its correlation with flap characteristics. Cancers (Basel).

[30] Nguyen A.T., Suami H., Hanasono M.M., Womack V.A., Wong F.C., Chang E.I. (2017). Long-term outcomes of the minimally invasive free vascularized omental lymphatic flap for the treatment of lymphedema. J Surg Oncol.

[31] Moon K.C., Kim H.K., Lee T.Y., You H.J., Kim D.W. (2022). Vascularized lymph node transfer for surgical treatments of upper versus lower extremity lymphedema. J Vasc Surg Venous Lymphat Disord.

